# Commentary: Enhanced Hemodynamic and Clinical Response to αCGRP in Migraine Patients—A TCD Study

**DOI:** 10.3389/fneur.2021.663818

**Published:** 2021-03-18

**Authors:** Claudia Altamura, Fabrizio Vernieri

**Affiliations:** Headache and Neurosonology Unit, Neurology, Università Campus Bio-Medico di Roma, Rome, Italy

**Keywords:** migraine, cerebral hemodynamics, calcitonin gene-related peptide, cortical spreading depolarization, ultrasound

Historically, meningeal vessel nociceptive sensitization has been a milestone of the migraine pathophysiology knowledge and the starting point of implementing new therapeutic strategies. Activated trigeminovascular terminals release peptides, which modulate pain and vascular activity (1). This neurogenic influence on cerebral hemodynamics is part of a very fine orchestral action with myogenic (i.e., autoregulation), endothelial (i.e., endothelial reactivity), and metabolic responses (i.e., vasomotor reactivity) (Figure 1). The astrocyte production of prostaglandins and nitric oxide (NO) responds to the neuronal firing (i.e., neurovascular coupling), mediating smaller intraparenchymal arterioles' dilatation. The neurogenic control of medium and small size arteries, on the other hand, occurs through the activation of sympathetic, parasympathetic, and sensory neurons. The last ones act secreting calcitonin gene-related peptide (CGRP), NO, serotonin, and Pituitary Adenylate Cyclase-Activating Polypeptide [PACAP] (2). Of these, CGRP is probably the most potent vasodilatory agent released in the cerebral circulation (3). Visočnik et al. (4) investigated by transcranial doppler the effect of the infusion of αCGRP on cerebral hemodynamics in migraine patients and healthy controls. Confirming what was observed by Lassen (5) in a placebo-controlled design, they observed a reduction in mean velocity of the middle cerebral artery (MCA) in both groups reflecting vaso-dilation. The relative contribution of cerebral vessels' dilation in determining migraine pain is much debated.

**Figure 1:**
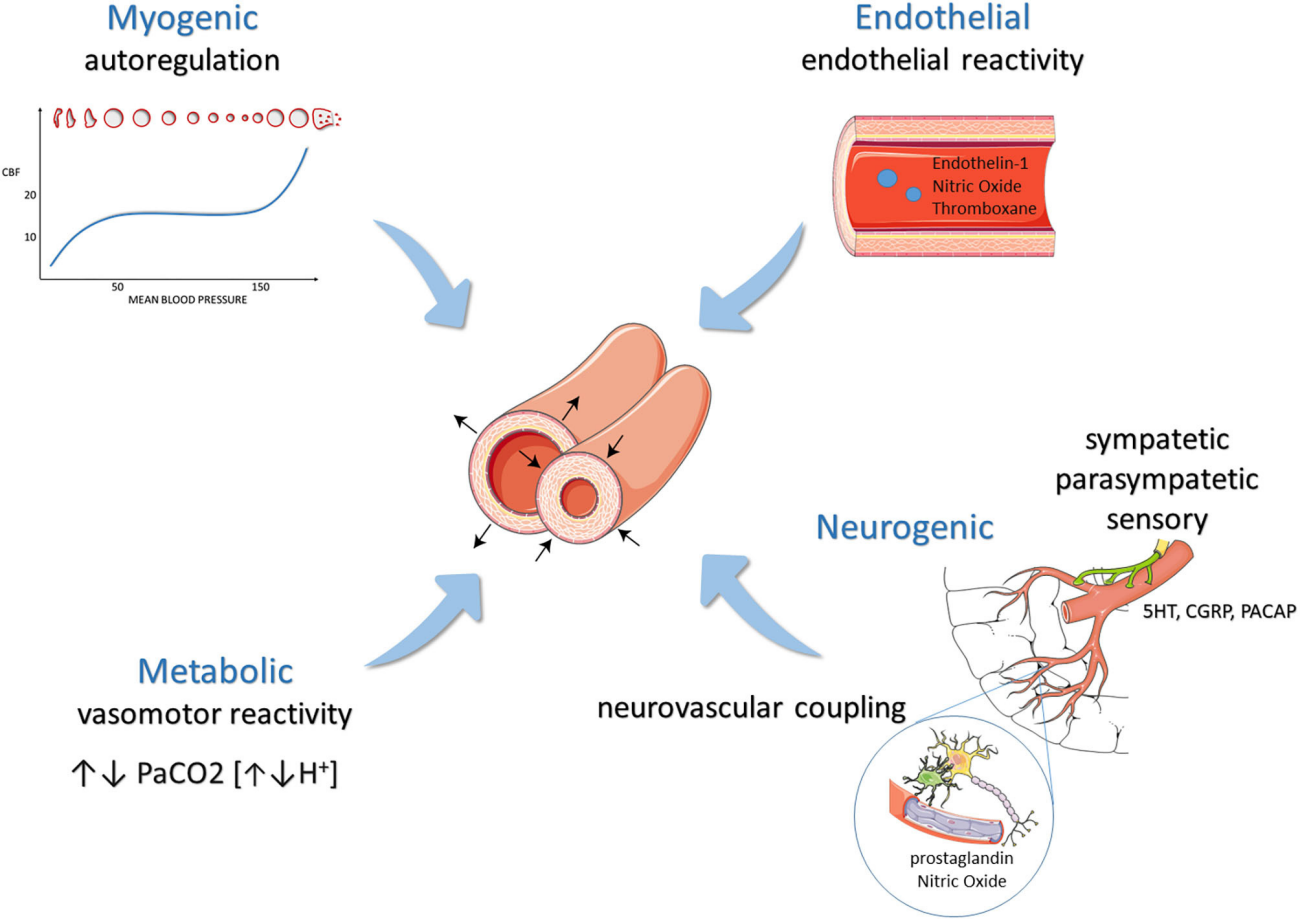
Modulation of cerebral hemodynamics: mechanisms balancing vasoconstriction and vasodilation.

Interestingly, not all cerebral vasodilatory agents (e.g., VIP, CO_2_) trigger migraine. Conversely, PACAP, which can dilate meningeal vessels but not MCA, can provoke headache in most subjects (6, 7) with a more pronounced response in migraine patients. These observations suggest that cerebral vessel dilation is an epiphenomenon of attacks rather than a trigger.

On the other hand, the MCA velocity reduction induced by αCGRP infusion was higher in migraineurs than controls, suggesting that they have a vascular tree more prone to sensitization (neurogenic modulation of hemodynamics) (4, 5). Similar hyperactive responses were observed under other conditions influencing hemodynamics. Nitric oxide super-sensitivity of the cerebral circulation has been consistently demonstrated in migraineurs (endothelial and parasympathetic control) (8). Similarly, migraine patients, in particular those with migraine with aura (MA), can display a higher response to hypercapnia (i.e., vasomotor reactivity, metabolic control) (9, 10). Finally, the vascular tree of MA patients is also more reactive to sensory inputs such as visual stimulation (i.e., neurovascular coupling) (11).

In the study by Visočnik and colleagues, mean arterial pressure was not modified during α-CGRP infusion, even if it increased significantly soon after only in migraine patients. This finding suggests that CGRP has no direct effect on systemic circulation. Accordingly, erenumab does not impair brachial flow-mediated dilation under physiological conditions in migraineurs without aura (12). On the other hand, peripheral vascular dysfunction was proposed in patients with migraine (13, 14). However, the studies addressing this topic reported highly conflicting findings, with some authors hypothesizing a hyperactive NO-supersensitive peripheral circulation in migraine (15).

It begs the question: if cerebral vessel dilation is just an epiphenomenon of migraine attacks, why is it promoted by the different mechanisms of hemodynamic control? The answer to this question would shed light on migraine pathophysiology and help understand the pathological link between migraine and stroke (16).

The monoclonal antibodies targeting the CGRP pathway are the first pharmacological therapies specifically developed for migraine prevention (17). These antibodies do not pass the blood-brain barrier for their high molecular weight; thus, their rapid efficacy in migraine prevention is mainly due to their peripheral action. Accordingly, Erenumab does not impair cerebral hemodynamics under physiological conditions in migraineurs without aura (12), nor the vasoactive response in isolated cranial arteries (18).

These observations suggest that CGRP is not involved in the control of hemodynamics under physiological conditions, reassuring the use of CGRP–targeted therapy in the clinical practice.

On the other hand, CGRP receptor antagonists seem to worsen cerebral ischemia in mice after MCA occlusion (19). Similarly, the autoregulatory vasodilation in response to hypotension was found attenuated by CGRP receptor desensitization in animal models (20). Altogether, this evidence points toward the hypothesis that CGRP can be called upon the need to rescue vasodilation when other compensatory mechanisms fail in extreme conditions.

If so, why is it released during migraine attacks? What would it be the physiological meaning and evolutionary advantage in migraine? To provide an answer to these questions, one could look at the relationship between cortical spreading depression (CSD) and CGRP (21, 22). CGRP seems not to trigger CSD while it is true the opposite (23). Genetic studies also support the primary role of cortical activation (i.e., CSD) in the migraine cascade, ultimately producing vessel dilation (24).

On the other hand, CGRP can modulate CSD propagation (23). Interestingly, cerebral blood flow can contra-regulate neural activity (the so-called vascular-neural coupling) (25). In this view, the physiological advantage of CGRP release would be to support the rapid wave of depolarization, increasing blood flow to meet the amplified metabolic demands. In this line, as opposed to the historical view of migraine as a neurovascular disorder, recent evidence bring light to the predominant role of the brain cortex and its metabolic demands due to aberrant plasticity and excitability as the pathophysiological substrate of the migraine cycle (26). Thus, more amply, the evolutionary role of CGRP is likely to prepare the brain to face stressful conditions (i.e., increased metabolic demand) via multiple mechanisms (27).

Finally, CGRP could counterbalance the action of other substances released during CSD with vasoconstrictive properties, such as endothelin-1 (28). In this line, CGRP related vasodilation can be considered a vascular adaptation to the metabolic and hemodynamic consequences of CSD.

This hypothesis would also explain the apparently paradoxical observation of a progressive improvement of cerebral hemodynamics along with migraine disease history (29, 30). These reports are also clinically reflected by the relative lower risk of stroke in MA patients with longer disease history, as in subjects with early-onset, compared with those with shorter disease history (31).

Altogether, these observations draw the picture of CRGP as “the goodfella” of cerebral hemodynamics, even if it is the ultimate guilty for migraine pain. Nevertheless, we are far from a full understanding of the relative role of CGRP in the physiology of cerebral hemodynamics and the physiopathology of migraine. It is particularly elusive the precise pathway conducting from neuro-excitability (32) to CGRP release (33). In the perspective of the development of new CGRP-targeted therapies for migraine, further research addressing this complex interrelationship is necessary, possibly combining neurophysiological and neurosonological investigations.

## Author Contributions

CA performed literature revision and drafted the manuscript. FV reviewed the manuscript content.

## Conflict of Interest

The authors declare that the research was conducted in the absence of any commercial or financial relationships that could be construed as a potential conflict of interest.
